# Frequency of Antimicrobial Resistance Genes in *Salmonella* From Brazil by *in silico* Whole-Genome Sequencing Analysis: An Overview of the Last Four Decades

**DOI:** 10.3389/fmicb.2020.01864

**Published:** 2020-08-07

**Authors:** Grazielle Lima Rodrigues, Pedro Panzenhagen, Rafaela Gomes Ferrari, Anamaria dos Santos, Vania Margaret Flosi Paschoalin, Carlos Adam Conte-Junior

**Affiliations:** ^1^Nucleus of Food Analysis (NAL), Laboratory for the Support of Technological Development (LADETEC), Chemistry Institute, Department of Chemistry, Federal University of Rio de Janeiro, Rio de Janeiro, Brazil; ^2^Food Science Graduate Program (PPGCAL), Chemistry Institute, Department of Chemistry, Federal University of Rio de Janeiro, Rio de Janeiro, Brazil; ^3^Health Surveillance Graduate Program (PPGVS), National Institute for Quality Control in Health (INCQS), Oswaldo Cruz Foundation, Rio de Janeiro, Brazil

**Keywords:** resistance genes, tetracycline, ciprofloxacin, antimicrobial surveillance, *gyrA*, *parC*, *tet*(A), *qnrE1*

## Abstract

*Salmonella* is a leading human pathogen and a significant public health concern worldwide. Massive food production and distribution have contributed to this pathogen dissemination, which, combined with antimicrobial resistance (AMR), creates new control challenges in food safety. The development of AMR is a natural phenomenon and can occur in the bacterial evolutionary process. However, the overuse and the misuse of antimicrobial drugs in humans and in animals have increased AMR selective pressure. In Brazil, there is an accuracy lack in AMR frequency in *Salmonella* because too many isolates are under-investigated for genetic and phenotypic AMR by the Brazilian health authorities and the research community. This underreporting situation makes the comprehension of the real level of *Salmonella* AMR in the country difficult. The present study aimed to use bioinformatics tools for a rapid *in silico* screening of the genetic antimicrobial resistance profile of *Salmonella* through whole-genome sequences (WGS). A total of 930 whole-genome sequences of *Salmonella* were retrieved from the public database of the National Biotechnology Information Center (NCBI). A total of 65 distinct resistance genes were detected, and the most frequent ones were *tet*(A), *sul2*, and *fosA7*. Nine point mutations were detected in total, and *parC* at the 57 position (threonine → serine) was the highest frequent substitution (26.7%, 249/930), followed by *gyrA* at the 83 position (serine → phenylalanine) (20.0%, 186/930) and at the 87 position (aspartic acid → asparagine) (15.7%, 146/930). The *in silico* prediction of resistance phenotype showed that 58.0% (540/930) of the strains can display a multidrug resistance (MDR) profile. Ciprofloxacin and nalidixic acid were the antimicrobial drugs with the highest frequency rates of the predicted phenotype resistance among the strains. The temporal analysis through the last four decades showed increased frequency rates of antimicrobial resistance genes and predicted resistance phenotypes in the 2000s and the 2010s when compared with the 1980s and 1990s. The results presented herein contributed significantly to the understanding of the strategic use of WGS associated with *in silico* analysis and the predictions for the determination of AMR in *Salmonella* from Brazil.

## Introduction

*Salmonella* is a frequent human pathogen, and salmonellosis is a global public health concern. Among the diarrheal and/or invasive agents, including bacteria, viruses, protozoa, helminths, and chemicals, *Salmonella* is among the 31 pathogens with a high capability of causing intestinal or systemic disease in humans (World Health Organization [WHO] (ed.), 2015). It is also the third cause of death among food-transmitted diseases (World Health Organization [WHO] (ed.), 2015). The massive production and the broad distribution of food have contributed to the rapid dissemination of this pathogen worldwide, which, combined with the presence of antimicrobial resistance (AMR) strains, creates new control challenges in food safety and public health on a global scale (Majowicz et al., 2010).

The emergence of AMR in bacteria may occur during the evolutionary process when cells accumulate random genetic mutations (resistance-mediating) in pre-existing genes and then transfer them *via* vertical gene transfer (Founou et al., 2016). The acquired resistance involves genetic exchanges within and between bacterial strains and species (Apata, 2009) and implies horizontal gene transfer with the acquisition of new antimicrobial resistance genes harbored on mobile genetic elements, such as plasmids, transposons, and integrons (Founou et al., 2016; Kudirkiene et al., 2018). Although the selection of antimicrobial-resistant bacteria is a natural process, the therapeutic use and the misuse of antimicrobial drugs in humans and animals have increased the selective pressure through the last decades (O’Neill, 2016). In addition, a large number of pathogenic bacteria, such as *Salmonella*, due to constant exposure to several different antimicrobial drugs, have recently been selected for resistance against one or more of these agents (dos Reis et al., 2018; Collignon and McEwen, 2019; Van Boeckel et al., 2019; Rabello et al., 2020). The current development lack of novel antimicrobials to replace the first-generation drugs brings the urgency to preserve the efficacy of existing drugs through the judicious use of antimicrobials. When the antimicrobials commonly used against the pathogenic bacteria are no longer effective, it is necessary to use drugs considered until the moment as “reserve” or “last resort” and these antimicrobials are often overpriced and/or can cause strong side effects (O’Neill, 2016).

In this context, it is important to acquire accurate information regarding the level of antimicrobial resistance as well as the data on antimicrobial use, employing harmonized strategies for sampling, management of data and metadata, and analysis. For example, the accurate knowledge of the resistance level present in the bacteria population associated with livestock animals is essential to plan strategies to control the overuse and the indiscriminate use of antimicrobials in animal production centers. As well known, instigating better practices in antimicrobial drug use avoids both the emergence and the spread of antimicrobial resistance in bacteria among multiple sources, like humans, animals, and the environment (Mackenzie et al., 2012; Collignon and McEwen, 2019). The surveillance of antimicrobial use is one of the most efficient tools to reduce the resistance dissemination and a valuable tool to measure its impact on both human and animal health (Johnson, 2015).

In Brazil, there is an accuracy lack in AMR frequency in *Salmonella* because too many isolates are under-investigated for genetic and phenotypic AMR by the Brazilian health authorities and the research community. This underreporting situation makes the comprehension of the real level of *Salmonella* AMR in the country difficult. Several national studies have focused on analyzing the phenotypic antimicrobial resistance in *Salmonella* (Dias de Oliveira et al., 2005; Campioni et al., 2012; Mattiello et al., 2015; Zishiri et al., 2016; Rodrigues et al., 2020), but few recent studies have focused on whole-genome sequence (WGS) analyses of resistant genes (Almeida et al., 2018; Monte et al., 2019a). Moreover, the current global trend is to use whole-genome sequencing in outbreak detection and surveillance routine of pathogenic bacteria (Didelot et al., 2012; Wilson, 2012; Leekitcharoenphon et al., 2014). Hence, including whole-genome sequencing for the surveillance of AMR genes in bacteria seems to be efficient as well. This study aims to investigate the presence of antimicrobial resistance genes in publicly available Brazilian *Salmonella* genomes from multiple-source isolated strains in the last four decades. To achieve this purpose, we combined the use of different bioinformatics software for a rapid *in silico* detection of resistance genes and also predicting the possibilities of a real phenotypic resistance in *Salmonella* from Brazil.

## Materials and Methods

### Data Collection

A total of 1,077 *Salmonella* whole-genome sequences were downloaded from the Sequence Read Archive (SRA) database in the National Biotechnology Information Center (NCBI) website. The genomes and their metadata information were retrieved for all SRA runs available as of July 2019 from Brazil. Four highly relevant metadata fields were originally kept from the source to standardize the contained terms and highlight the value of the sequence record: serovar, collection date, strain name, and isolation source. To find all genomes available from Brazil, we first search through the keyword “*Salmonella* and Brazil,” and then we use the “Run Selector” SRA tool to select only the genomes sequenced by the Illumina platform. In addition to maintaining the original information for each record, curated fields were created and can be accessed in Supplementary Table S1.

### Genome Assembly and Quality Filtering

Raw Illumina paired-end reads were downloaded and assembled as reported in the following pipeline. The sequences downloaded from NCBI in SRA format were performed using the software SRAtoolkit 2.9.0, with the –prefetch command option specified. These downloaded sequence files were further converted to fastq format with the option –fastq-dump specified. The genomes with less than 80% of reads with minimum “Phred Quality Score 30” detected by the FastQC software were discarded. The software Trimmomatic 0.36 (ILLUMINACLIP: TruSeq3-PE.fa:2:30:12 LEADING:3 TRAILING:3 MAXINFO:40:0.999 MINLEN:36) (Bolger et al., 2014) was used to cut adapters, Illumina-specific sequences, and other fragments with low quality. *De novo* genome assembly was performed by using the SPAdes 3.10.0 software with –careful option activated (Bankevich et al., 2012). Finally, the software QUAST 5.0.2 (Gurevich et al., 2013) was used, with standard command options, as the quality assessment tool for evaluating and comparing genome assemblies. The quality filtering criteria were the following: assemblies with a size <4 or >6 Mb failed the size criteria. The assemblies were excluded if they have a final number of contigs >500. The assemblies with N50 value <10 kb were also excluded.

### Data Standardization

To reduce variability and standardize the strain isolation source, they were clustered in five groups according to the origin similarities and host proximity. The clinical-animal group included *Salmonella* genomes isolated from feces, biological fluid or tissue, blood, lymph nodes, and others. The clinical-human group included isolates from blood, brain abscess, feces, coproculture, hip secretion, and others. In the food group, the non-animal-based foods, such as beans, papaya, tomato, potatoes, vegetables in general, and others were considered. The animal-based food group included foods that have chicken, eggs, pork, beef, milk, or seafood in the composition or raw cuts. The environment group included animal box swab, flock environment, truck after cleaning, chicken cage after cleaning, disposable shoe covers of the farm’s workers, and slaughterhouse environment. These five source groups, after genome assemblies and quality filtering exclusion, comprised 930 out of the 1,077 *Salmonella* genomes initially downloaded from NCBI. The number of genomes per group were as follows: animal-based food, *n* = 374; clinical-human, *n* = 249; clinical-animal, *n* = 133; environment, *n* = 110; and food, *n* = 64 (Supplementary Table S1). Also, to reduce the variabilities in the isolation date of bacterial strains, we only considered information about the year of isolation, which was from the last four decades ranging from 1980 to 2018.

### *In silico* Analysis

The assembled genomes were uploaded to SISTR^1^ (Yoshida et al., 2016) using the application programming interface, and the resulting serovar predictions were compared to the reported serovar on the NCBI record (Supplementary Table S1). In all cases of disagreements between SISTR predictions and the informed serovar by NCBI metadata, we considered the results of SISTR prediction with quality check approved. All samples with failure in SISTR quality check were discarded and not used in our study.

For the *in silico* identification of antimicrobial resistance genes and the prediction of phenotypic antimicrobial resistance, we used the software STARAMR 0.7.1^2^. The software scanned bacterial genome contigs against the ResFinder (Zankari et al., 2012) and PointFinder (Zankari et al., 2017) databases and compiled a summary report of the detected antimicrobial resistance genes. Database scanning was settled for the minimum DNA identity of 95% and the minimum DNA coverage of 60% for all genome alignments. Additionally, STARAMR compiled the possible predicted phenotypes of the microbiological resistance, but not the clinical resistance, with support from the NARMS/CIPARS Molecular Working Group that continually is improving the prediction accuracy.

### Statistics and Temporal Analyses

The overall frequency was calculated by the ratio between each type of antimicrobial resistance (the acquired antimicrobial resistance gene or chromosomal point mutations or predicted AMR phenotype) and the total number of genomes assessed in the study. The frequency heat maps were constructed to represent each antimicrobial resistance mechanism in all sources studied (clinical-animal, clinical-human, food, animal-based food, or environment) and/or in the four most frequent serovars (*S.* Enteritidis, *S.* Typhimurium, *S.* Heidelberg, and *S.* Dublin). The R software^3^ was used, combined with the Complex Heatmap Package, from Bioconductor, to construct the frequency heat maps (Gu et al., 2016).

Three main graphs were constructed to display the temporal analysis of the nine more frequent resistance genes detected, the three more frequent chromosomal mutations, and the predicted AMR resistance within the 930 genomes of the genus *Salmonella*. Moreover, 12 individual supplementary graphs were constructed to display the temporal analysis of the nine more frequent resistance genes detected and the three more frequent chromosomal mutations within the four more frequent *Salmonella* serovars (*S.* Dublin, *S.* Enteritidis, *S.* Heidelberg, and *S.* Typhimurium). Finally, one last supplementary temporal graph was constructed to display the temporal frequency of the four more frequent *Salmonella* serovars along with all serovars detected through the decades. For all these analyses, we considered the information about the year of isolation as supplied in the original records of the NCBI metadata. To reduce the variability in the isolation date of the bacterial strains, we only considered information about the year of isolation, which was from the last four decades ranging from 1980 to 2018. The frequencies of the genus *Salmonella* or each *Salmonella* serovar were calculated by the ratio between each type of antimicrobial resistance and the total number of genomes from strains collected in each decade. The serovars’ temporal frequency was calculated by the ratio between each serovar and the total number of genomes from the strains collected in each decade.

## Results

### *Salmonella* Serovars

Among the 930 complete genomes of *Salmonella enterica* analyzed, 46 distinct serovars were detected *in silico* (Supplementary Table S1). The serovars were *Salmonella* Enteritidis (34.7%, *n* = 323), *S.* Typhimurium (20.4%, *n* = 190), *S.* Heidelberg (15.3%, *n* = 142), *S.* Dublin (11.9%, *n* = 111), and others (17.6%, *n* = 164) (*S*. Abaetetuba, *S*. Abony, *S*. Adelaide, *S*. Agona, *S*. Albany, *S*. Anatum, *S*. Braenderup, *S*. Brandenburg, *S*. Cerro, *S*. Cubana, *S*. Gallinarum, *S*. Give, *S*. Grumpensis, *S*. I 1,4,[5],12:d:-, *S*. I 1,4,[5],12:i:-, *S*. Idikan, *S*. Infantis, *S*. Javiana, *S*. Kentucky, *S*. Livingstone, *S*. London, *S*. Madelia, *S*. Mbandaka, *S*. Minnesota, *S*. Montevideo, *S*. Muenchen, *S*. Muenster, *S*. Newport, *S*. Ohio, *S*. Oranienburg, *S*. Orion, *S*. Ouakam, *S*. Panama, *S*. Poona, *S*. Rissen, *S*. Saintpaul, *S*. Sandiego, *S*. Schwarzengrund, *S*. Senftenberg, *S*. Tennessee, *S*. Westminster, and *S*. Yoruba) (Supplementary Table S1).

**FIGURE 1:**
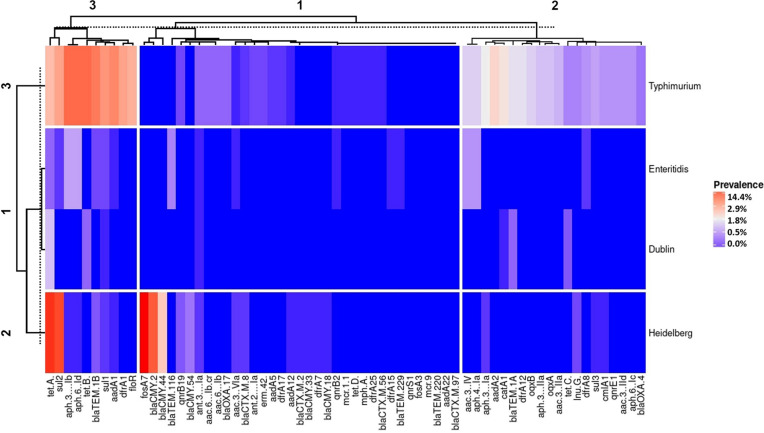
Heat map with the antimicrobial resistance gene frequency of *Salmonella* isolated from Brazil by the most frequent serovars deposited in the National Biotechnology Information Center- Sequence Read Archive database.

The four most *in silico* detected serovars (*S.* Typhimurium, *S.* Enteritidis, *S.* Dublin, and *S.* Heidelberg) were clustered in three different groups according to their similarity between the resistance gene frequency (vertical clustering in Figure 1). Cluster 1 displayed *Salmonella* Enteritidis and *S.* Dublin as having a similar genetic resistance profile and lower frequency rates of resistance genes. Cluster 2 was composed by *Salmonella* Heidelberg, which displayed high frequency rates of resistance genes only for *tet*(A), *fosA7*, *sul2*, and *bla*_CMY–__2_. Lastly, cluster 3 displayed that *Salmonella* Typhimurium has an intermediate to high frequency rate of resistance genes and showed the highest diversity of antimicrobial resistance genes (horizontal clustering in Figure 1).

### *Salmonella* Resistome

A total of 65 different resistance genes were found among the 930 *Salmonella* genomes analyzed (Supplementary Table S2). In general, the highest frequency of AMR genes detected was against aminoglycosides (40.7%, *n* = 379). This class of antimicrobials also showed the largest gene diversity, comprising 18 different genes conferring resistance to this class [*aac(3)-IIa*, *aac(3)-IId, aac(3)-IV*, *aac(3)-VIa*, *aac(6’)-Ib*, *aadA1*, *aadA2*, *aadA5*, *aadA12, aadA22, ant(2”)-Ia*, *ant(3”)-Ia*, *aph(3”)-Ib*, *aph(3’)-IIa*, *aph(3’)-Ia*, *aph(4)-Ia*, *aph(6)-Ic*, and *aph(6)-Id*]. The second highest frequency class of AMR genes detected was tetracycline (28.9%, *n* = 269), with four different genes [*tet*(A), *tet*(B), *tet*(C), and *tet*(D)]. The gene *tet*(A) was the most frequent, detected in 20.1% genomes (187/930). β-Lactamases producer genes also presented a high frequency (24.6%, *n* = 229), with 16 different genes (*bla*_CMY–__2_, *bla*_CMY–__33_, *bla*_CMY–__44_, *bla*_CMY–__18_, *bla*_CMY–__54_, *bla*_CTX–M–__2_, *bla*_*C*__TX–M–__8_, *bla_*C*_*_TX–M–__56_, *bla_*C*_*_TX–M–__97_, *bla*_OXA–__17_, *bla*_OXA–__4_, *bla*_TEM–__220_, *bla*_TEM–__116_, *bla*_TEM–__229_, *bla*_TEM–__1__*A*_, and *bla*_TEM–__1__*B*_). The sulfonamide resistance genes (23.6%, *n* = 220) were *sul1*, *sul2*, and *sul3*. Genes conferring resistance to chloramphenicol (*catA1* and *cmlA1*), trimethoprim (*dfrA12*, *dfrA15*, *dfrA17*, *dfrA1*, *dfrA25*, *dfrA8*, and *dfrA7*), florfenicol (*floR*), fosfomycin (*fosA3* and *fosA7*), lincosamide [*lnu*(G)], colistin (*mcr*-1.1 and *mcr*-9), macrolide [*mph*(A)], fluoroquinolone [*oqxA*, *oqxB*, and *aac(6’)-Ib-cr*], quinolone (*qnrB19*, *qnrB2*, *qnrE1*, and *qnrS1*), and macrolide/lincosamides/streptogramins [*erm*(42)] were also found but in lower frequencies (Supplementary Table S1). Finally, besides *tet*(A), the genes *sul2* and *fosA7* also were considered as highly frequent, being detected in 16.9% (157/930) and 14.9% (139/930) of the WGS, respectively (Supplementary Table S1).

**FIGURE 2:**
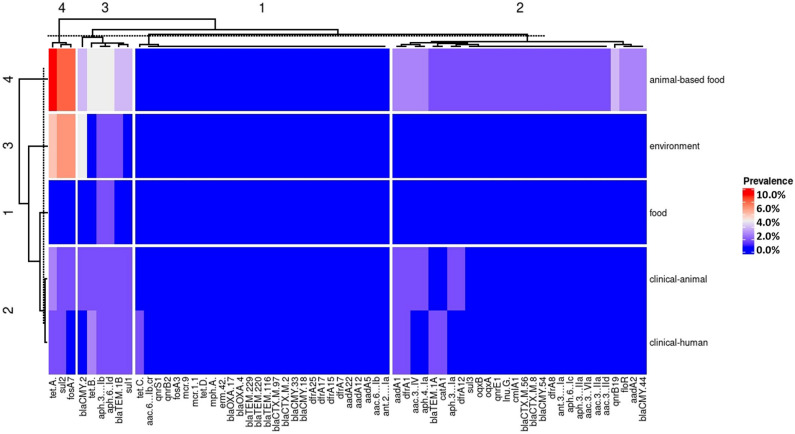
Heat map with the antimicrobial resistance gene frequency of *Salmonella* isolated from Brazil by the five strain’s source groups.

Strains isolated from clinical-animal and clinical-human sources presented the most similar genetic AMR profile, as shown in heat map cluster 2 (left side of Figure 2). Strains isolated from food and environment sources displayed a similar genetic AMR profile (heat map clusters 1 and 3), and strains isolated from animal-based food showed a quite distinct genetic AMR profile with the higher frequency values, as displayed by heat map cluster 4 (left side of Figure 2).

Resistance genes were clustered in four different groups according to their frequency similarities (upper side of Figure 2). Cluster 1 joined the lower frequency rates of antimicrobial resistance genes: [*tet*(C), *aac(6’)-Ib-cr*, *qnrS1*, *qnrB2*, *fosA3*, *mcr*-9, *tet*(D), *mph*(A), *erm*(42), *bla*_OXA–__17_, *bla*_OXA–__4_, *bla*_TEM–__229_, *bla*_TEM–__220_, *bla*_TEM–__116_, *bla*_CTX–M–__2_, *bla*_CMY–__33_, *bla*_CMY–__18_, *dfrA25*, *dfrA15*, *dfrA17*, *dfrA7*, *aadA22*, *aadA12*, *aadA5*, *aac(6’)-Ib*, and *ant(2”)-Ia*]. Cluster 2 was composed by genes showing lower frequency rates in strains isolated from clinical-human, clinical-animal, environment, and food sources and intermediate rates from animal-based food [*aadA1*, *dfrA1*, *aac(3)-IV*, *aph(4)-Ia*, *bla*_TEM–__1__*A*_, *catA1*, *aph(3’)-Ia*, *dfrA12*, *sul3*, *oqxB, oqxA*, *qnrE1*, *lnu*(G), *cmlA1*, *bla*_*C*__TX–M–__56_, *bla*_*C*__TX–M–__8_, *bla*_CMY–__54_, *dfrA8*, *ant(3”)-Ia*, *aph(6)-Ic*, *aph(3’)-IIa*, *aac(3)-IId*, *qnrB19*, *floR*, *aadA2*, and *bla*_CMY–__44_]. Cluster 3 was composed solely by genes displaying intermediate frequency rates such as *bla*_CMY–__2_, *tet*(B), *aph(3”)-Ib*, *aph(6)-Id*, *bla*_TEM–__1__*B*_, and *sul1*. Lastly, cluster 4 joined genes that showed the highest frequency rates in strains isolated from animal-based food and environment [*tet*(A), *sul2*, and *fosA7*] (upper side of Figure 2).

### *Salmonella* Chromosomal Mutation Conferring AMR

In the 930 WGS analyzed for chromosomal mutations, we identified nine distinct mutations that would result from an amino acid substitution (Supplementary Table S1). In *gyrA*, five distinct amino acid substitutions were found [three at the 87 position (aspartic acid → glycine or asparagine or tyrosine) and two at the 83 position (serine → phenylalanine and tyrosine)]. In *gyrB*, two distinct amino acid substitutions were found [at the 466 and 464 positions (glutamic acid → aspartic acid and serine → phenylalanine, respectively)]. In *pmrA* and *parC*, only one amino acid substitution was found in each [at the 53 and 57 positions (glycine → glutamic acid and threonine → serine, respectively)]. The three top amino acid substitutions that may result in resistance against nalidixic acid and ciprofloxacin were in *parC* at the 57 position (threonine → serine) with 26.7% (249/930), followed by *gyrA* at the 83 position (serine → phenylalanine) with 20.0% (186/930), and at the 87 position (aspartic acid → asparagine) with 15.7% (146/930) of frequency. A single genome presented a mutation that may result in colistin resistance (in *pmrA* at the 53 position glycine → glutamic acid). All the remaining WGS point mutations detected may result in resistance to ciprofloxacin and/or nalidixic acid.

**FIGURE 3:**
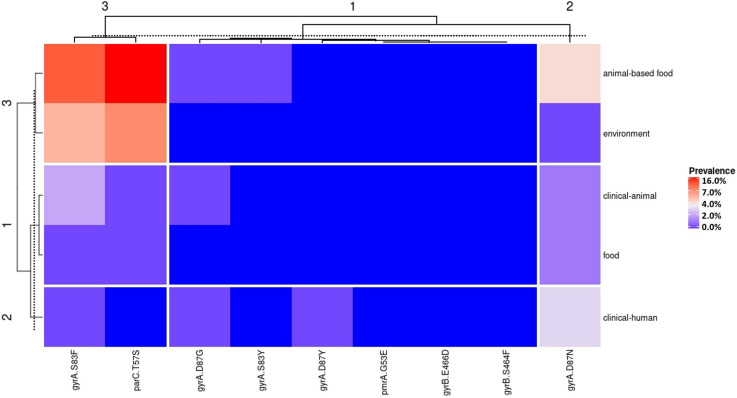
Heat map with the frequency of chromosomal mutation conferring antimicrobial resistance in *Salmonella* isolated from Brazil by the five strain’s source groups.

The mutation in *parC* at the position 57 (threonine → serine) was the most frequent in WGS from animal-based food and environmental sources; *gyrA* at the position 83 (serine serine → phenylalanine) was the most frequent in the clinical-animal source; *gyrA* at the position 87 (aspartic acid → asparagine) was the most frequent in food and clinical-human sources (Figure 3). In a general overview, strains isolated from clinical-animal, clinical-human, and food sources presented a closely related genetic AMR profile, as shown in heat map clusters 1 and 2 (left side of Figure 3). Strains isolated from animal-based food and environment sources displayed another closely related genetic AMR profile (heat map cluster 3 on the left side of Figure 3).

### *Salmonella* Predicted AMR Phenotypes

The *in silico* prediction analysis of the resistance phenotype from the 930 *Salmonella* WGS showed that 46.6% (434/930) of the strains are possible pan-susceptible to all analyzed antimicrobial classes and 59.2% (551/930) might present phenotypical drug resistance to one or more antimicrobial classes (Supplementary Table S1). Furthermore, 58.0% (540/930) of strains have a predicted potential to display a MDR phenotypical profile, described as resistant to at least three classes of antimicrobial agents (Magiorakos et al., 2012). Ciprofloxacin and nalidixic acid showed the highest rates of possible phenotypical resistance among the strains, with 45.6% (425/930) and 42.3% (394/930) of predicted resistance in all strain genomes, respectively. Tetracycline, ampicillin, and sulfisoxazole also showed high rates of predicted resistance, with 27.1% (252/930), 24.2% (225/930), and 23.0% (214/930), respectively.

**FIGURE 4:**
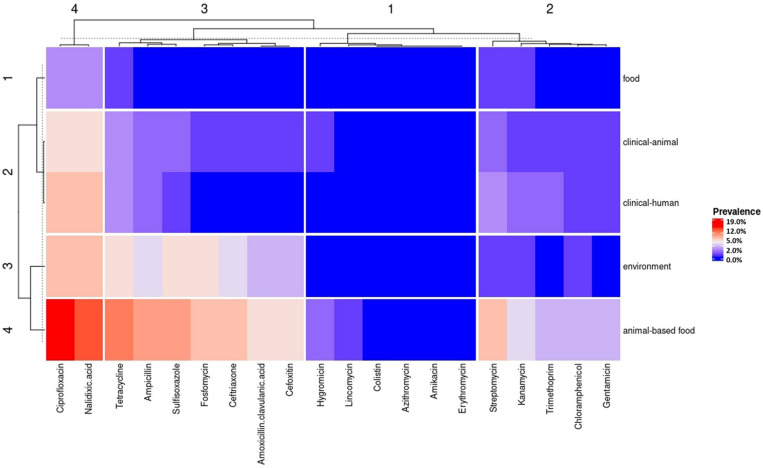
Heat map with the predicted antimicrobial resistance phenotype frequency by the antimicrobial drug in *Salmonella* from the five strain’s source groups.

Antimicrobial drugs that have a predicted phenotypic resistance were grouped into four clusters according to their similarity between the sources analyzed (upper side of Figure 4). Cluster 1 joined the lower frequency rates of the predicted phenotype resistance in all sources (hygromycin, lincomycin, colistin, azithromycin, amikacin, and erythromycin). Cluster 2 was composed of antimicrobials with intermediate frequency rates of the predicted phenotype resistance (streptomycin, kanamycin, trimethoprim, chloramphenicol, and gentamicin) (upper side of Figure 4). Cluster 3 was composed by antimicrobials with intermediate frequency rates of the predicted phenotype resistance in clinical-animal and clinical-human sources and high frequency rates in the environment and animal-based food sources (tetracycline, ampicillin, sulfisoxazole, fosfomycin, ceftriaxone, amoxicillin/clavulanic acid, and cefoxitin). Finally, cluster 4 joined the highest frequency rates of the predicted phenotype resistance (ciprofloxacin and nalidixic acid) (upper side of Figure 4).

The animal-based food source group comprised the highest number of predicted MDR strains (16.5%, 154/930), with 66 different profiles. The highest frequency of predicted MDR profile was against six antimicrobial classes (penicillin, fosfomycin, quinolone, tetracycline, sulfonamide, and β-lactams). The environment, clinical-animal, clinical-human, and food sources comprised the predicted MDR strains, with 6.6% (61/930), 2.9% (27/930), 2.6% (24/930), and 0.4% (4/930) of frequency, respectively (Supplementary Table S1).

### Overall Temporal Analysis

For the temporal analysis, we used all of the 928 WGS, of which the year of isolation was provided in the metadata (Supplementary Table S1). The frequencies were split and analyzed through four decades. In the decade of 1980, 62 WGS were analyzed; in 1990, 151 WGS were analyzed; in 2000, 232 WGS were analyzed; and in 2010, 483 WGS were analyzed.

**FIGURE 5:**
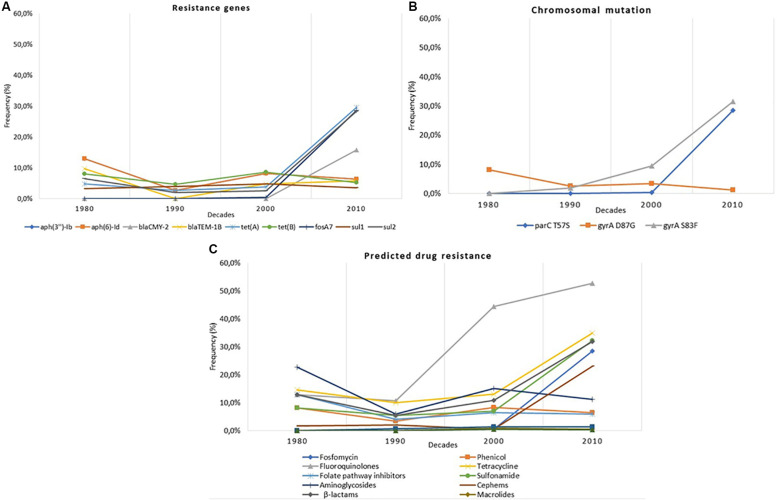
**(A)** Temporal distribution of antimicrobial resistance genes within *Salmonella* whole-genome sequences (WGS) in Brazil from the 1980s to the 2010s. **(B)** Temporal distribution of the chromosomal mutations within *Salmonella* WGS in Brazil from the 1980s to the 2010s. **(C)** Temporal distribution of the predicted antimicrobial resistance phenotype by antimicrobial class within *Salmonella* WGS in Brazil from the 1980s to the 2010s.

The temporal analysis was on the nine highest-frequency resistance genes. The study displayed that the frequency rates of the genes *tet*(A), *sul2*, *fosA7*, and *bla*_CMY–__2_ increased in the Brazilian *Salmonella* spp. strains after the 2000s. The frequency rates of the genes *tet*(B), *aph(3”)-Ib*, *aph(6)-Id*, *bla*_TEM–__1__*B*_, and *sul1* remained constant throughout the last four decades (Figure 5A).

Concerning chromosomal mutation, the temporal analysis was based on the three highest-frequency chromosomal mutations detected in the study. The frequency rates of *gyrA* at the 87 position (aspartic acid → glycine) decreased very less throughout the last decades. On the other hand, *gyrA* at the 83 position (serine → phenylalanine) and *parC* at the 57 position (threonine → serine) considerably increased after the 1990s (Figure 5B).

Overall, the temporal analysis of the *in silico* prediction of phenotypic AMR within the Brazilian strains displayed a similar pattern as the genetic profile. Until the 2000s, the majority of the strains displayed constant frequency rates of the predicted phenotypical resistance. However, the predicted phenotypical resistance against fluoroquinolones considerably increased after the 1990s, and the predicted resistance against tetracycline, sulfonamide, and β-lactams increased after the 2000s (Figure 5C).

The supplementary temporal analysis revealed that the increase of some antimicrobial genes and chromosomal mutations is strongly related to some of the most frequent *Salmonella* serovars. For example, the increase of *bla*_*CMY–*__2_, *bla*_*TEM–*__1__*B*_, *tet*(A), *fosA7*, *sul2*, and *parC* at the 57 position (threonine → serine) and *gyrA* at the 83 position (serine → phenylalanine) is closely related to the increased frequency of *Salmonella* Heidelberg (Supplementary Figures S1A–M). Moreover, *aph(3”)-Ib, aph(6)-Id*, *tet*(B), *sul1*, and *gyrA* at the 87 position (aspartic acid → glycine) are closely related to resistance in *Salmonella* Typhimurium (Supplementary Figures S1A–M). Finally, the numbers of *Salmonella* Dublin, *S*. Enteritidis, and *S*. Typhimurium decreased its WGS frequency after the 1990s and the 2000s, respectively. However, *Salmonella* Heidelberg and other serovars increased their WGS frequency after the 2000s (Supplementary Figure S1M).

## Discussion

The increasing frequency of the predicted AMR profiles among *S. enterica* reflected on the collected genomes over the last four decades represents a national public health concern, mainly due to the frequent cross-contamination between food, clinical, and environmental pathogens. A cross-sectoral global action is required to control better the AMR *Salmonella* spreading to different sources. The use of novel tools as next-generation sequencing (NGS) technologies has been beneficial in detecting those bacteria. However, NGS produces a high amount of complex raw data that have to be analyzed by appropriate bioinformatics tools to turn the raw reads into meaningful data (Tang et al., 2017). Because the cost of genome sequencing is decreasing, it is becoming practical to use *in silico* tools to predict bacterial AMR genotypes and phenotypes directly from the WGS data. Although we still need to isolate bacteria using traditional microbiology techniques in order to obtain the genomes, the advancement in whole genome sequencing and the application of online tools for real-time detection of AMR determinants are essential to identify control and prevention strategies to combat the increasing threat of AMR (Hendriksen et al., 2019). Accessible tools and DNA sequence data are expanding, which will allow establishing global pathogen surveillance and AMR tracking based on genomics (Hendriksen et al., 2019). To detect AMR phenotypes from WGS, it is first necessary to understand the genetic AMR profile present among strains and the impact that these variations have on the phenotype. Herein the NGS and the bioinformatics were combined to perform a genomic associated study using WGS to enhance the investigation and the surveillance.

The present study, as far as we know, is the first one performed in Brazil to get an update and accurate information from raw data on *S. enterica* antimicrobial resistance in the last four decades. Instead of NCBI GenBank or RefSeq assembly database, the SRA database was chosen for sequence downloads due to some particularities. First, raw sequence download comes along with per base quality score, which allowed the screening for high-quality genome sequencing, increasing the reliability of the final data. Second, the SRA database provides a higher number of genomes from *Salmonella* strains found in Brazil in comparison with both RefSeq and GenBank assembly. Finally, through the SRA Run selector tool, essential details such as the geolocation, sequencing platform, and serovar were easily and quickly filtered in order to cure the metadata.

Although the number of deposited WGS of *S. enterica* in Brazil is not as wide as North America’s and Europe’s number of deposits, we recovered a considerable number of 930 high-quality sequences. *Salmonella* Enteritidis (34.7%, 323/930) and *S.* Typhimurium (20.4%, 190/930) were the most commonly found serovars among the WGS deposited from Brazil. *Salmonella* Enteritidis and *S.* Typhimurium are the most common serovars reported in human sources in Latin America between 2001 and 2014 (Hendriksen et al., 2011; Quesada et al., 2016), in the United States in 2016 (Centers for Disease Control and Prevention [CDC], 2018), in member countries of the European Union in 2017 (European Food Safety Authority [EFSA], and European Centre for Disease Prevention, and Control [ECDC], 2018), and in the majority of the researches and the studies regarding *Salmonella* around the world (Asif et al., 2017; Borges et al., 2017; Campioni et al., 2017; Muvhali et al., 2017; Utrarachkij et al., 2017; Zhang et al., 2017; Magdy et al., 2019). The high presence of these serovars among the WGS deposited from Brazil may be related to the fact that the majority of researches about *Salmonella* in Brazil also concerns *S.* Enteritidis and *S.* Typhimurium and likewise worldwide (Campioni et al., 2017; Almeida et al., 2018; Panzenhagen et al., 2018a, b; Ritter et al., 2019).

We found a high diversity of resistance genes (*n* = 65) by the *in silico* analysis of *Salmonella* WGS. The highest frequency of antimicrobial resistance genes was against the aminoglycoside class, which also showed the highest diversity of resistance genes (*n* = 18). The *aac(3)-IIa*, *aac(6’)-Ib*, *ant(2”)-Ia*, *aac(3)-IId*, *aac(3)-IV*, *aac(3)-VIa*, and *ant(3”)-Ia* genes belong to the aminoglycoside N-acetyltransferase group, which are a chromosome-encoded superfamily of aminoglycoside acetyltransferase proteins (Salipante and Hall, 2003; Ramirez and Tolmasky, 2010). In Brazil, a pioneer study regarding antimicrobial resistance in 748 *Salmonella* strains revealed higher rates of resistance against aminoglycosides (streptomycin) in animals (87.7%) and animal feed isolates (91.3%) (Campos and Hofer, 1989). This high streptomycin resistance rates in *Salmonella* from animal and animal feed can be evidence of possible aminoglycoside use as growth promoters in food-producing animals in Brazil since the drug’s discovery in 1944. Moreover, aminoglycoside’s broad spectrum of antimicrobial activity and the frequent success in synergy with other antimicrobials (van Hoek et al., 2011) can also justify their constant use and high resistance rates in *Salmonella* over the years in Brazil. The *aadA1*, *aadA2*, *aadA5*, and *aadA12* AMR genes also confer resistance against the aminoglycoside class, more specifically to streptomycin/spectinomycin. This is a common resistance profile found in *Salmonella* (Ramirez and Tolmasky, 2010; Lopes et al., 2014, 2016). All these genes are situated on gene cassettes in classes 1 or 2 integrons or make part of the *Salmonella* genomic island (SGI) 1 or genomic island 2 (SGI2) and usually are associated with MDR gene clusters (Michael and Schwarz, 2016). Those genomic features lead to a more incident horizontal transmission of AMR in bacteria.

Resistance genes *tet*(A) and *sul2* that may confer resistance against tetracycline and sulfonamide, respectively, were also highly frequent among the analyzed WGS, with 20.1% (187/930) and 16.9% (157/930) frequency, respectively (Figure 2). These resistance genes also displayed the highest frequency in *Salmonella* from the animal-based food sources. The high frequency of these resistance genes in animal-based food sources can be explained by the routine use of tetracycline and sulfonamide as growth promoters in poultry and swine husbandry in Brazil (Dias de Oliveira et al., 2005; dos Santos et al., 2009). In agreement with our previous study regarding the phenotypical resistance of *Salmonella* for the last 30 years in Brazil (Rodrigues et al., 2020), both tetracycline and sulfonamide displayed high frequency rates of antimicrobial resistance among *Salmonella* isolated from swine. These results corroborate with the evidence that the indiscriminate use of antimicrobials in animal husbandry is increasing the antimicrobial resistance in *Salmonella* (Van Boeckel et al., 2019).

The *fosA7* resistance gene may confer resistance against fosfomycin, and it was firstly identified in *Salmonella* in 2017 (Rehman et al., 2017). According to Rehman et al. (2017), in *Salmonella*, the gene *fosA7* is exclusively located on the chromosome. However, it is potentially transferable *via* horizontal gene transfer. Currently, the frequency of *fosA7* among *Salmonella* serovars is limited to a few serovars, with *Salmonella* Heidelberg as the most common carrier (Rehman et al., 2017). In agreement, our results showed a frequency of 15.5% (144/930) *Salmonella* WGS with the *fosA7* resistance gene, and the only serovar with *fosA7* occurrence was *S*. Heidelberg. The potential horizontal gene transfer of *fosA7* creates a great concern of this gene spreading to other bacteria due to the increased use of fosfomycin in both clinical treatments and in animal husbandry, making it necessary to control the use of this antimicrobial as well as to monitor the spread of fosfomycin resistance in bacteria.

Quinolone and fluoroquinolone resistance usually results from a point mutation, predominantly in the conserved quinolone resistance-determining regions (QRDR) involved in DNA binding (Varughese et al., 2018). The amino acid substitutions modify the housekeeping genes, such as the prime targets DNA gyrase (*gyrA* and *gyrB*) and topoisomerase IV (*parC* and *parE*), making them less susceptible to quinolone binding (Maka and Popowska, 2016). Particularly, the mutations in the *gyrA* gene seem to be the leading cause of most quinolone resistance in *Salmonella* (Seminati et al., 2005). Our *in silico* analyses highlighted eight distinct amino acid substitutions that may confer increases in the minimum inhibitory concentration to nalidixic acid and ciprofloxacin. Although *Salmonella* harboring QRDR mutations can cause infections more challenging to treat, they are not typically horizontally transmissible, which limits the rate and the range of spreading of these resistances among the bacterial population (Tyson et al., 2017). Nowadays, the plasmid-mediated quinolone resistance (PMQR) is an effective threat to the therapeutic use of quinolone (Rodríguez-Martínez et al., 2016). The plasmid quinolone resistance *qnr* genes include a set of genes *qnrA*, *qnrB*, *qnrC*, *qnrD*, *qnrS*, and *qnrE*, in which the latest has been circulating in South America since 2000 (Strahilevitz et al., 2009; Almeida et al., 2018; Panzenhagen et al., 2018a; Monte et al., 2019b; Soares et al., 2019).

Despite the wide variety of existing PMQR, only *qnrB19*, *qnrB2*, *qnrS1*, and *qnrE1* were detected in all WGS analyzed. Interestingly, *qnrE1* was detected only in *Salmonella* Typhimurium WGS obtained from isolates from animal-based food and clinical-animal sources. However, previous studies have reported the presence of *qnrE1* in other *Salmonella* serovars in Brazil (Soares et al., 2019). Evidence from the phylogenetic reconstruction of *qnr* genes analyzing the *qnrE1* environment showed that this PMQR was probably mobilized by ISEcp1 from the *Enterobacter* spp. chromosome to the *Klebsiella pneumoniae* plasmids (Albornoz et al., 2017). Although in our results and in the results provided by Monte et al. (2019b) only *S*. Typhimurium showed the *qnrE1* gene, Soares et al. (2019) described the occurrence of *qnrE1* in IncM1 plasmids in *Salmonella* Newport, *S*. Enteritidis, and *S*. Infantis recovered from human clinical sources in Brazil. The genetic plasticity of the *qnrE1* plasmid highlights the horizontal transmission capability between bacterial plasmids from several species, serovars, and environmental sources (Monte et al., 2019a). Nevertheless, the higher frequency of *S*. Typhimurium carrying the *qnrE1* detected here and by Monte et al. (2019a) highlights the possibility of clonal spread from a common ancestor of *S*. Typhimurium carrying the *qnrE1* plasmid in Brazil.

The altered locus of *pmrA* (*polymyxin resistance A*) displays a decreased susceptibility to certain cationic agents that bind to lipopolysaccharide (Helena Makela et al., 1978), and it was firstly identified in *Salmonella* Typhimurium mutants that displayed increased resistance to polymyxins B (Helena Makela et al., 1978; Roland et al., 1993). This polymyxin resistance mechanism is chromosomally mediated and cannot be horizontally transmissible (Liu et al., 2016). However, nowadays, the new plasmid-mediated colistin resistance mechanism (*mcr*) is globally distributed (Wang et al., 2018). Our *in silico* analysis identified a single *Salmonella* WGS presenting *pmrA* mutation (at the 53 position, glycine → glutamic acid), which may result in resistance to polymyxin. Moreover, three analyzed *Salmonella* WGS showed *mcr* genes (one WGS showed *mcr*-1.1 and the other two showed *mcr*-9). The announcement of the first report of *mcr* genes in *Salmonella* from Brazil has been recently published (Rau et al., 2018; Moreno et al., 2019) and highlights the intercontinental spread of this plasmidial gene. Currently, colistin is not the first choice to treat human infections caused by *Salmonella*, and the development of clinical colistin resistance is apparently non-relevant. However, phenotypical colistin resistance has been observed in *S. enterica* and *E. coli* strains from food-producing animals in Brazil (Morales et al., 2012), and when resistance determinants are inserted in genetic mobile elements (e.g., *mcr*-like genes), they quickly can be horizontally transferred to other bacterial species of animal and human origin. The presence of *mcr*-like genes should not be neglected in zoonotic pathogens such as *Salmonella*. Our results highlight the importance of the controlled use of colistin in livestock and agriculture to curb the spread of colistin resistance since, in humans, colistin is used as a last-resort antimicrobial, treating extensively antimicrobial-resistant pathogens (Zavascki et al., 2007).

In 1998, Brazilian authorities have restricted the use of sulfonamides and tetracyclines only to clinical treatment in animal husbandry, banning their use as growth promoters (Voss-Rech et al., 2017). Even with these prevention measures, our temporal analysis revealed that the frequency rates of the genes *tet*(A) and *sul2* and the predicted AMR against sulfonamide and tetracycline notably increased during the last decades, with the highest increase after the 2010s (Figure 5).

The significant incidence of extended-spectrum cephalosporin (ESC) resistance has been noted in different *Enterobacteriaceae* and has become an important public health concern worldwide (Livermore, 2012). One of the most important reasons for resistance to ESC in *Enterobacteriaceae* is *AmpC* β-lactamase (Batchelor et al., 2005). The resistance plasmid gene *bla*_*CMY–*__2_ encodes an *AmpC*-type β-lactamase, which hydrolyzes the third-generation cephalosporins (Bauernfeind et al., 1996; Zhao et al., 2001). Although the third generation of cephalosporins is classified as “critically important” for human health (World Health Organization [WHO], 2017), ceftiofur is usually used in day 1 chicks, associated with Marek’s vaccine to prevent the disease in broilers (Webster, 2009). In Canada, the use of ceftiofur in poultry production was responsible for the increase of resistant *Salmonella* Heidelberg (Lucie Dutil et al., 2010). Our results displayed that the *bla*_*CMY–*__2_ gene frequency in *Salmonella* Heidelberg is also high in Brazil (Figure 1). Although in scientific publications the frequent occurrence of ESBLs (typically CTX-M-2) in broiler raw meat products imported to Europe from Brazil is documented, human health threats related to these ESBLs seem to be seldomly discussed in Brazil until now (Bokma et al., 2014). The use of antimicrobial in animal production is evidenced by the antimicrobial drug residue surveillance program, highly emphasized by both the Brazilian Ministry of Agriculture, Livestock and Supply (MAPA) and the integrations (Bokma et al., 2014). The evidence found here likewise endorses the hypothesis that the overuse of ceftiofur in the Brazilian poultry production may be the cause of the increase of the *bla*CMY-2 frequency in *Salmonella* Heidelberg. We also demonstrated that the *bla*CMY-2 gene has a frequency increase, as well as the predicted AMR phenotype against β-lactams after the 2010s (Figure 5). The broad-spectrum characteristics of the third-generation cephalosporins may result in an overuse of this antimicrobial class since they can treat a wider range of infections and may be used in clinical human treatment as well as in animal production.

Animal-based food was the most common source of the predicted antimicrobial-resistant *Salmonella* (Figure 2). The strains isolated from this source also comprised the highest number of predicted MDR phenotype resistance (16.5%, 154/9,310), with the greatest diversity of predicted MDR profiles (*n* = 66). The emergence of MDR among *Salmonella* contaminating the animal-based food is a global public health concern. Although intensive antibiotic therapy can select associated AMR pathogens and the misuse and the overuse of antimicrobials in animal husbandry may cause resistance in bacteria in the treated host, the correlation between resistance in animal-based food and the antimicrobials used in husbandry is poorly determined (McDermott et al., 2018). The emergence of AMR in bacteria can compromise the effectiveness of antimicrobials in human therapeutic treatments as well as favor the resistance propagation through multiple sources. Here we bring out the importance of the surveillance of antimicrobial use, especially in animal husbandry, as an essential tool to control and curb the spread of antimicrobial resistance.

## Conclusion

It is of concern that little epidemiological surveillance data of AMR is publicly available in Brazil. The establishment of strategies to monitor the genetic and the phenotypic AMR is of utmost importance to supply the underestimation of AMR frequency, particularly in pathogenic bacteria from different sources such as food, animal-based food, clinical-human, clinical-animal, and environment. The results presented herein contributed significantly to the understanding of the strategic use of WGS associated with *in silico* analysis and the predictions for the determination of AMR in *Salmonella* from Brazil. Point frequency surveys and prediction analysis are imperfect and far from the best approach for epidemiological surveillance networks. However, in the absence of systematic surveillance, *in silico* studies seem to be useful to guide interventions against AMR increase. Furthermore, more detailed studies on resistance genes carried by mobile genetic elements are necessary to reveal how the AMR interchange occurs between different sources.

## Data Availability Statement

All datasets presented in this study are included in the article/Supplementary Material.

## Author Contributions

PP conceived and designed the study. GR, PP, and AS retrieved data and performed bioinformatics and temporal analysis. GR performed the statistical analysis and wrote the first draft of the manuscript. All other authors contributed to manuscript revision and read and approved the submitted version.

## Conflict of Interest

The authors declare that the research was conducted in the absence of any commercial or financial relationships that could be construed as a potential conflict of interest.

## Footnotes

1 https://lfz.corefacility.ca/sistr-app/

2 https://github.com/phac-nml/staramr

3 http://www.r-project.org

## Supplementary Material

FIGURE S1 Temporal distribution of the most frequent resistance genes and chromosomal mutations *aph(3”)-Ib* **(A)**, *aph(6)-Id* **(B)**, *bla*_*CMY–2*_ **(C)**, *bla*_*TEM–1B*_ **(D)**, *tet*(A) **(E)**, *tet*(B) **(F)**, *fosA* **(G)**, *sul1* **(H)**, *sul2* **(I)**, *parC* at the 57 position threonine → serine **(J)**, *gyrA* at the 87 position aspartic acid → glycine **(K)**, *gyrA* at the 83 position serine → phenylalanine **(L)** within the four most frequent *Salmonella* serovars (*S.* Dublin, *S.* Enteritidis, *S.* Heidelberg, and *S.* Typhimurium) and *Salmonella* spp. **(M)** in Brazil from the 1980s to the 2010s.

TABLE S1 National Biotechnology Information Center original metadata information associated with the detection of antimicrobial resistance genes and the phenotypic resistance prediction of *Salmonella* genomes within the five strain’s source groups. The numbers 0 and 1 mean the absence and the presence, respectively.

TABLE S2 Distribution frequency of the 65 antimicrobial resistance genes analyzed in *Salmonella* genomes separated in the five strain’s source groups.
