# Supplementary figures and images for: Frequency of Antimicrobial Resistance Genes in Salmonella From Brazil by in silico Whole-Genome Sequencing Analysis: An Overview of the Last Four Decades
Source: Front Microbiol. 2020 Aug 7;11:1864. doi: 10.3389/fmicb.2020.01864 (PMC7426471; doi:10.3389/fmicb.2020.01864)

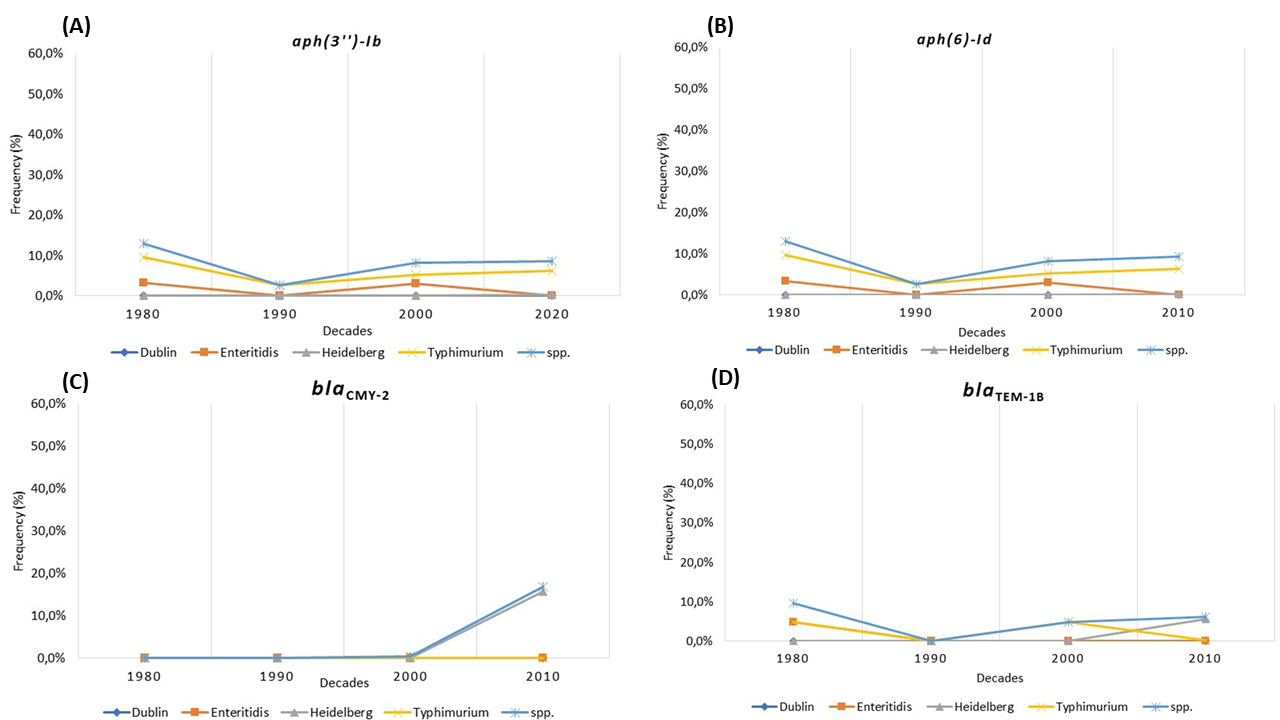

Supplement: FIGURE S1 — Temporal distribution of the most frequent resistance genes and chromosomal mutations aph(3”)-Ib (A), aph(6)-Id (B), blaCMY–2 (C), blaTEM–1B (D), tet(A) (E), tet(B) (F), fosA (G), sul1 (H), sul2 (I), parC at the 57 position threonine → serine (J), gyrA at the 87 position aspartic acid → glycine (K), gyrA at the 83 position serine → phenylalanine (L) within the four most frequent Salmonella serovars (S. Dublin, S. Enteritidis, S. Heidelberg, and S. Typhimurium) and Salmonella spp. (M) in Brazil from the 1980s to the 2010s. [file Image_1.TIF]

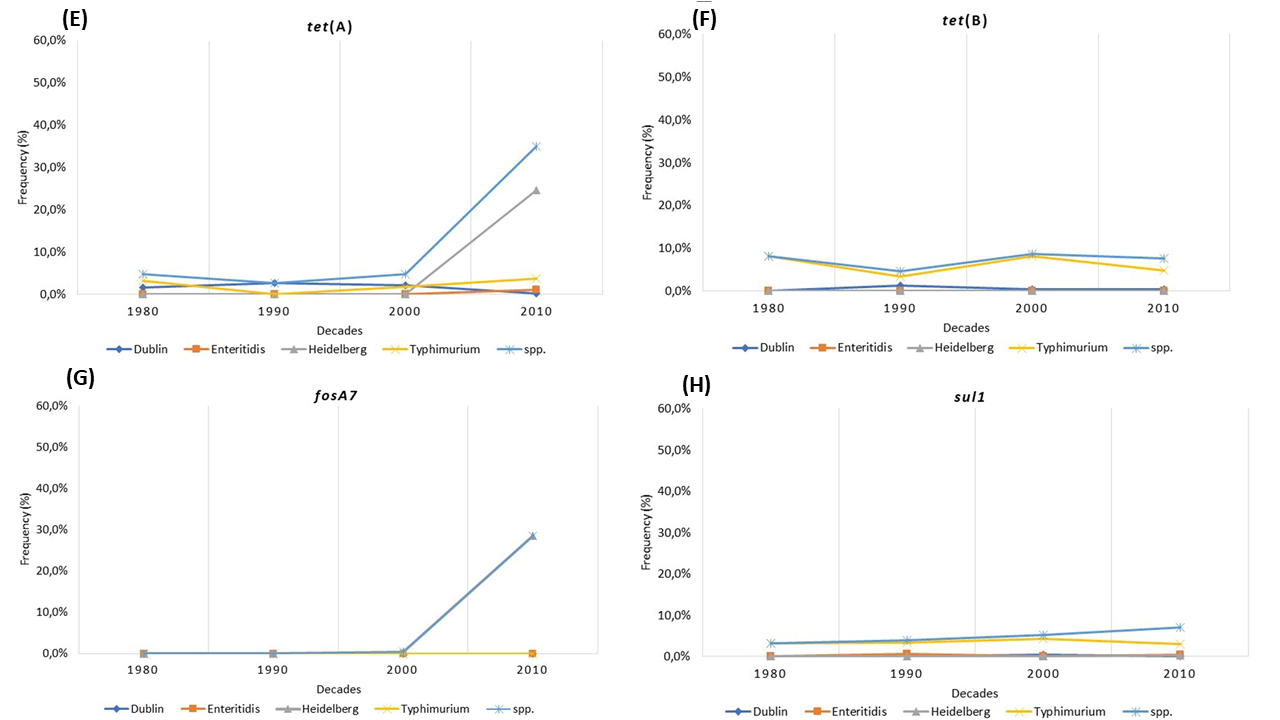

Supplement: Supplementary file 2 [file Image_2.TIF]

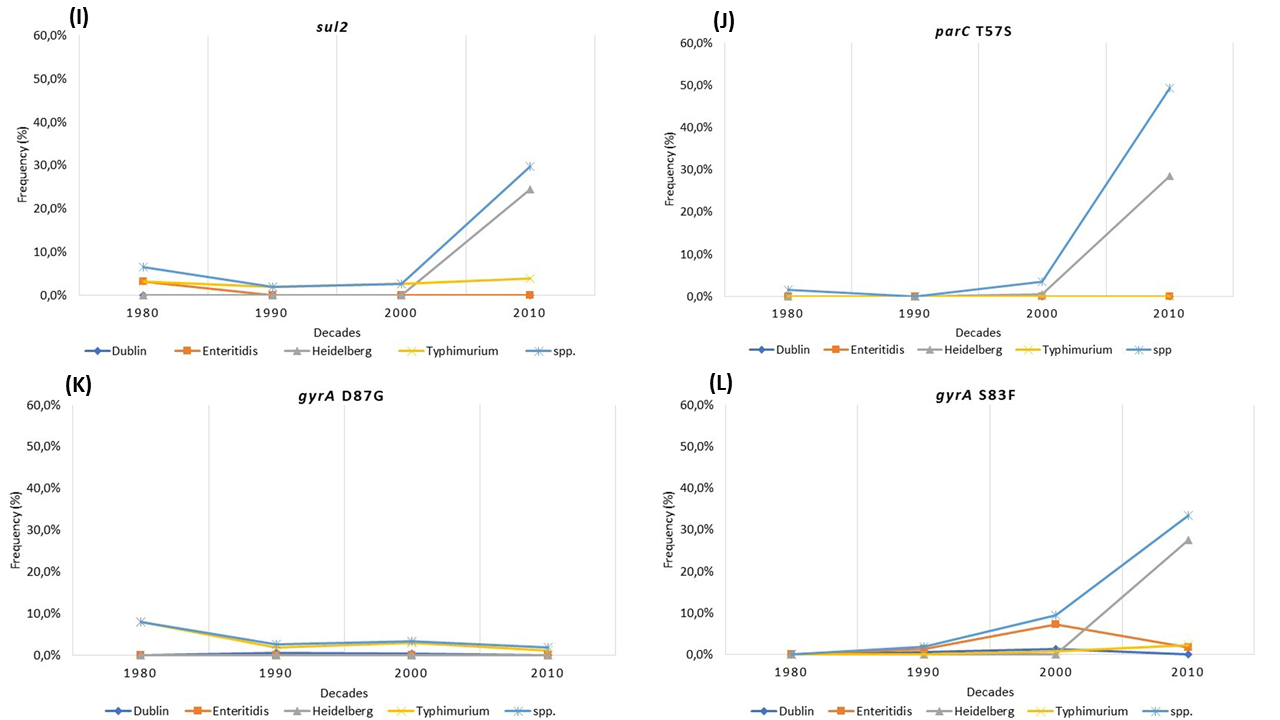

Supplement: Supplementary file 3 [file Image_3.TIF]

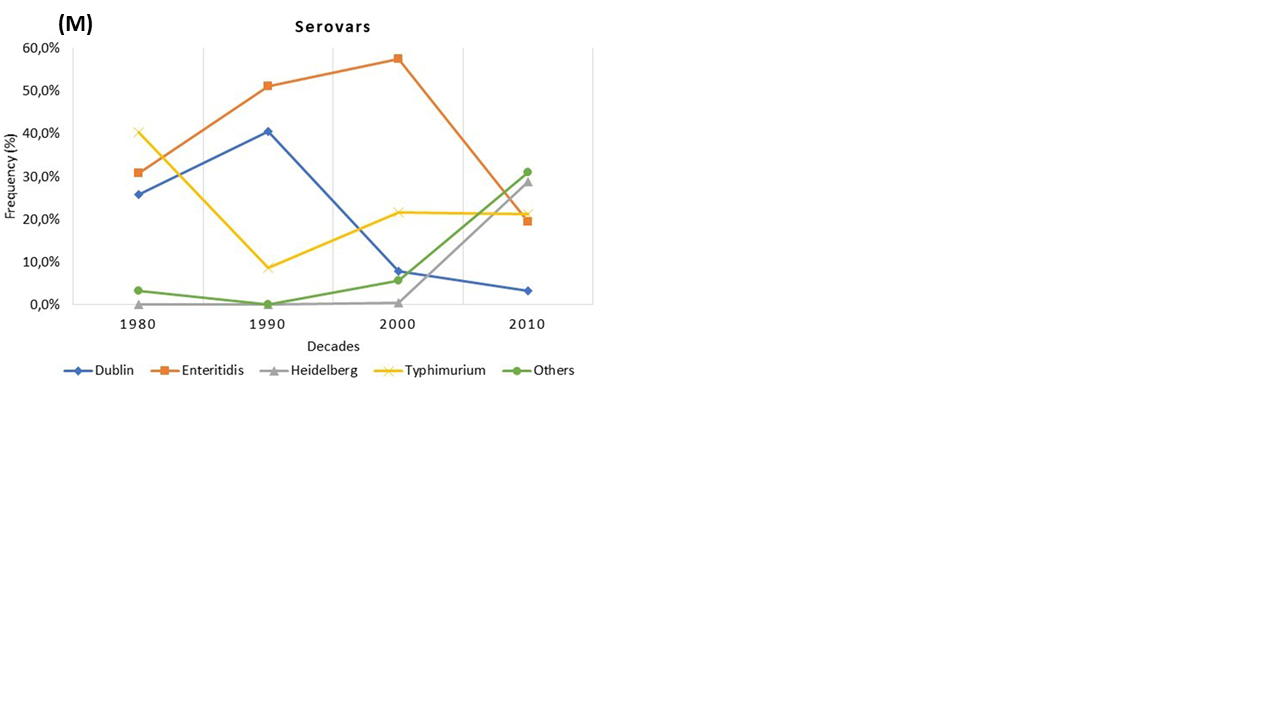

Supplement: Supplementary file 4 [file Image_4.TIF]
